# Practice of CYP450 genotyping and phenotyping in children in a real-life setting

**DOI:** 10.3389/fphar.2023.1130100

**Published:** 2023-02-27

**Authors:** Frédérique Rodieux, Youssef Daali, Victoria Rollason, Caroline F. Samer, Kuntheavy Ing Lorenzini

**Affiliations:** ^1^ Division of Clinical Pharmacology and Toxicology, Department of Anesthesiology, Pharmacology, Emergency Medicine and Intensive care, Geneva University Hospitals, Geneva, Switzerland; ^2^ School of Pharmaceutical Sciences, University of Geneva, Geneva, Switzerland; ^3^ Faculty of Medicine, University of Geneva, Geneva, Switzerland

**Keywords:** children, pharmacogenetics, genotyping, phenotyping, CYP450

## Abstract

Pharmacokinetics varies widely between children. Many factors play an important role in this variability, such as ontogeny, pharmacogenetics, gender, comorbidities, and drug-drug interactions. Significant work has already been done in adults to understand the impact of genetic polymorphisms on drug-metabolizing enzyme activity and drug response. Data remain poor in children due to ontogeny that impacts genotyping-phenotyping correlation and the difficulty enrolling children in prospective studies. Our study aimed to describe the use of cytochromes P450 (CYP) phenotyping and/or genotyping tests in children in a real-life setting and assess the correlation between the genotype and the phenotype. We reviewed the results of tests performed between January 2005 and December 2020. Fifty-two children were genotyped and/or phenotyped. Four patients were excluded from the present analysis as they only underwent ABCB1 genotyping, without CYP testing. Of the remainder, 18 underwent simultaneous CYP genotyping and phenotyping, while 17 underwent CYP genotyping only, and 13 underwent CYP phenotyping only. In all cases, investigations were performed after the following situations: insufficient clinical response to treatment, low plasma concentrations, and adverse drug reactions (ADR). The vast majority of cases were related to immunosuppressive or antipsychotic therapy. Genotyping and/or phenotyping explained or contributed to the aforementioned clinical events in 56% of cases. The correlation between the genotype and the phenotype showed variability depending on the assessed cytochrome. In several cases, the phenotype did not correspond to the genotype because of comedications. In conclusion, there is clearly value in guiding drug based on CYP activity in children.

## Introduction

Children differ from adults not only in their growth in height and weight, but also in the rapid physiological, maturative, and developmental changes known as ontogeny. This results in a very heterogeneous population with wide variability in drug response (Kearns et al., 2003).

Physiological developmental changes occurring in children affects all pharmacokinetic processes, from absorption and distribution to metabolism and excretion, as well as the pharmacodynamic response (Kearns et al., 2003; Allegaert and van den Anker, 2019). The impact on drug metabolizing enzyme (DME) activity is significant. Other factors that play an important role in the variability of DME activity and thus drug response in children are, like in adults, pharmacogenetics, gender, comorbidities, and exogenous factors such as drug-drug interactions (DDI).

Cytochromes P450 (CYP) enzymes play a major role in drug metabolism and are among the most extensively studied DME in both adults and peadiatric populations. Drug-drug interactions involving CYP enzymes have received considerable attention for years (Ogu and Maxa, 2000; Nimmagadda et al., 2020). The mechanisms of CYP inhibition and induction are now fairly well understood. Knowing whether prescribed drugs act as enzyme substrates, inducers, or inhibitors can prevent the occurrence of drug toxicity or inefficacy. In adults, many studies have already been conducted to determine the impact of individual CYP genetic variations on drug response (Zhou et al., 2009; Samer et al., 2013; Goh et al., 2017; Manikandan and Nagini, 2018). Increasingly, expert consensus guidelines for dose adjustment or drug selection based on CYP polymorphisms are being developed to improve the likelihood of positive outcomes, reducing both adverse drug reactions (ADR) and treatment failure (Swen et al., 2008; Relling and Klein, 2011; Swen et al., 2011; Volpi et al., 2018; Relling et al., 2020). Existing approaches to predict CYP activity are genotyping and phenotyping procedures. While CYP genotyping is based on DNA analysis and prediction of enzyme activity from the identified alleles, phenotyping uses the administration and measurement of enzyme-specific probes to determine metabolic activity *in vivo*. The Geneva cocktail has been successfully used for years to measure the patients’ phenotype in our institution. This phenotyping cocktail is composed of seven low-dose specific probes for six different CYP and for P-glycoprotein (P-gp) (Bosilkovska et al., 2014; Rollason et al., 2020b).

In children, defining CYP activity and its impact on drug response is challenging. When considering existing approaches to determine DME activity, genotyping is limited due developmental regulation of gene expression, and phenotyping is made difficult by the necessity to administer a non-toxic exogenous probe for this population and the need to develop very sensitive analytical methods due to the small dose that can be administered (Magliocco and Daali, 2020). Our study aimed to describe the use of CYP phenotyping and/or genotyping tests in children in a real-world setting, including practical issues related to this specific population, and the benefits for patient management. We also assessed the correlation between the predicted phenotype (based on the genotype) and the measured phenotype.

## Materials and methods

### Patients and setting

This study is a paediatric subpart of three previously published studies that included children and adults (Lloret-Linares et al., 2017; Rollason et al., 2020a; Ing Lorenzini et al., 2021). The studies were approved by the Ethics Commission of the Canton of Geneva, Geneva (Switzerland) (study numbers: 15–225, 15–080 and 14–244) (Lloret-Linares et al., 2017; Rollason et al., 2020a; Ing Lorenzini et al., 2021).

In this study, we evaluated in- and out-paediatric patients, aged 0–17 years, whose CYP genotyping or phenotyping tests were performed between January 2005 and December 2020 as part of their routine care. In Switzerland, genetic analyses fall under the Federal Act on Human Genetic Testing and as such may be performed only after having obtained written informed consent from parents/legal guardians (Baumann et al., 2017) (FOPH, 2018). We reviewed the results of the tests performed during the study period and the reason for these investigations.

### Genotyping

Genotyping was performed by Geneva University Hospitals’ laboratory of molecular oncology and pharmacogenomics as described previously (Rollason et al., 2020a; Ing Lorenzini et al., 2021). Genotyping techniques have considerably evolved over the last few years. In brief, until 2007, only CYP2D6 genotyping was performed, by real-time polymerase chain reaction (PCR). The following alleles were detected: *3, *4, *5, *6, *35, *41, and duplications. From 2007, genotyping was performed on the AmpliChip CYP450 test from Roche. For CYP2D6, 33 alleles could be analyzed. For CYP2C9, *2 and *3 could be detected while for CYP2C19, the detectable alleles were *2 and *17. From 2017, we used Next-Generation Sequencing (NGS) technologies with the pharmacogenomics panel from ThermoFisher. CYP2D6 gene copy number was determined by qPCR on LC480 (Roche) using CNV Exon 9 Hs00010001_cn and CNV Intron 6 Hs04502391_cn probes for CYP2D6 (Life Technologies, with RNAse P gene used as reference gene). We used AlleleTyper Software (Thermo Fisher Scientific) to translate genetic pattern information from genotyping (Single-nucleotide polymorphisms—SNP) and copy number assay to their standardized allele name or star (*) allele nomenclature.

Prediction of the phenotype based on the identified alleles was performed as described in our previous article (Ing Lorenzini et al., 2021) by using the Pharmacogene Variation (PharmVar) Consortium database (Gaedigk et al., 2018; Gaedigk et al., 2019). We classified patients into one of the following categories: poor metabolizer (PM), intermediate metabolizer (IM), normal metabolizer (NM), rapid metabolizer (RM), and ultra-rapid metabolizer (UM).

### Phenotyping

Phenotyping was performed using exogenous oral enzyme-specific probes; namely, subtherapeutic doses of caffeine, bupropion, flurbiprofen, omeprazole, dextromethorphan, midazolam, and fexofenadine for CYP1A2, CYP2B6, CYP2C9, CYP2C19, CYP2D6, CYP3A4/5, and P-glycoprotein activity assessment, respectively. The probes were administered orally on an empty stomach, either in the form of the Geneva cocktail (phenotyping cocktail approach) as described elsewhere (Bosilkovska et al., 2014) or by administering a specific targeted probe (selective phenotyping). In selective phenotyping situations, the same probes as in the Geneva cocktail were used; in children under 40 kg, the doses were adapted on a case-by-case basis to be subtherapeutics. In rare specific situations, the CYP3A/5 phenotype was measured by blood sampling during midazolam treatment. Phenotype determination was based on the metabolite to parent drug metabolic ratio (MR) measured on capillary or venous blood samples. Until 2012, CYP2D6 phenotyping was performed in urine as previously described (Rebsamen et al., 2009). The capillary route was preferred when the child did not have peripheral venous access. Venous blood was used when there was functional venous access or concomitant blood sampling. Based on their MR, we classified patients as PM, IM, NM, or UM (Rebsamen et al., 2009; Bosilkovska et al., 2014).

### Genotype-phenotype correlation

Based on the identified alleles on genotyping, the patients were attributed to a predicted phenotype category. Based on the measured metabolic ratio on phenotyping, the patients were attributed to a measured phenotype. We concluded to *concordance* when the measured phenotype was equal to the predicted phenotype. We concluded to *non-concordance* when the measured and predicted phenotypes were not equivalent. In these cases, we assessed the role of comedication and eventually concluded to drug-induced phenoconversion.

### Clinical data

We collected the main reason for genotyping/phenotyping, demographic data, and concomitant treatments. The reason for genotyping/phenotyping was one of the following: ADR/high drug levels, inefficacy/low drug levels, DDI, International Normalized Ratio (INR) variation, prescription (preemptive testing), and others. Based on the clinical pharmacology and toxicology consultation, two clinical pharmacologists concluded whether or not the results of the genotypic/phenotypic investigation explained the clinical situation.

### Statistical analysis

We used descriptive statistics to report our data. We described categorical variables with frequencies, and continuous variables with means (± standard deviation). We used the SPSS^®^ software package, version 25 (IBM corporation, Armonk, NY, United States) to perform the analyses. We concluded that genotype and phenotype were concordant when the measured phenotype corresponded to the predicted phenotype based on the alleles present. The cause of the non-concordance, including the presence of DDI, was investigated.

## Results

A total of 52 children underwent genotype or phenotype testing during the study period. Since our study focused on genotype-phenotype concordance, four patients were excluded from this analysis as they were only tested for ABCB1 due to an inability to assess concordance with phenotype for this gene. The mean age of the remaining 48 patients was 9.7 (± 5.4) years (range: One month to 17 years). Of the 48 patients, 17 (35%) underwent genotyping only, 13 (23%) underwent phenotyping only, and 18 (42%) underwent simultaneous CYP genotyping and phenotyping.

The mean age of the 17 children who were only genotyped was 8.3 ± 6.0 years (range: 0–16). Six of them were four years old or younger at the time of the investigation. We were unable to identify the reasons why phenotyping was not performed.

The mean age of the 13 children who were only phenotyped was 11.8 ± 4.1 years. Except for one child aged 1 year, all were 9 years or older. We were unable to identify the reasons why only phenotyping was performed.

A total of 18 children underwent simultaneous CYP genotyping and phenotyping. Seventeen had the same CYP isoenzyme simultaneously genotyped and phenotyped, while one patient had CYP2D6 genotyped but CYP2C9 phenotyped. The distribution of the predicted phenotype based on the genotype is presented in Table 1. We assessed the genotype-phenotype correlation in the 17 patients (mean age: 9.3 ± 5.4 years, range: 1–17). The concordance rates were highly variable, ranging from 80% for CYP2C9 to 33% for CYP3A5 (Table 2). In about half of the non-concordant cases for CYP2C19 and CYP3A5, this could be explained by a drug interaction (drug-induced phenoconversion) that either increased or decreased CYP activity (Table 3). For CYP2D6, almost all non-concordant cases (5/6) had increased activity of the measured phenotype compared to the genotype-based predicted phenotype. However, there is no known exogenous inducer of CYP2D6.

**TABLE 1:** CYP predicted phenotype based on the identified alleles in patients with simultaneous genotyping and phenotyping.

Isoenzyme	*n* ^a^		PM^b^	IM^b^	NM^b^	RM/UM^b^
CYP1A2	0	Numbers of children (%)	–	–	–	–
		Genotype	–	–	–	–
CYP2B6	0	Numbers of children (%)	–	–	–	–
		Genotype	–	–	–	–
CYP2C9	5	Numbers of children (%)	–	–	5 (100)	–
		Genotype	–	–	*1/*1	–
CYP2C19	8	Numbers of children (%)	–	1 (12.5)	4 (50)	3 (37.5)
		Genotype	–	*1/*2	*1/*1	*1/*17, *17/*17
CYP2D6	12	Numbers of children (%)	–	4 (33.3)	8 (66.6)	–
		Genotype	–	*1/*5, *1/*4, *5/*41	*1/*1, *2/*41, *1/*2A, 1/*35	–
CYP3A5	3	Numbers of children (%)	2 (66.6)	1 (33.3)	–	–
		Genotype	*3/*3	*1/*3	–	–

^a^ Number of cases that underwent simultaneous genotyping and phenotyping for the concerned isoenzyme; poor metabolizer (PM), intermediate metabolizer (IM), normal metabolizer (NM), rapid metabolizer (RM), and ultra-rapid metabolizer (UM).

^b^ Number (percentage).

**TABLE 2:** Concordance between the predicted and measured phenotype.

	*N* total^a^	-	*n* (%)	Details	
				Predicted phenotype	Measured phenotype
CYP2C9	5	Concordant (%)	4 (80)	NM (*n* = 4)	NM (*n* = 4)
		Non-concordant (%)	1 (20)	NM (*n* = 1)	UM (*n* = 1)
CYP2C19	8	Concordant (%)	3 (37.5)	RM (*n* = 1)	RM (*n* = 1)
				NM (*n* = 2)	NM (*n* = 2)
		Non-concordant (%)	5 (63.5)	IM (*n* = 1)	NM (*n* = 1)
				RM (*n* = 1)	NM (*n* = 1)
				NM (*n* = 1)	PM (*n* = 1)
				NM (*n* = 1)	UM (*n* = 1)
				UM (*n* = 1)	IM (*n* = 1)
CYP2D6	12	Concordant (%)	6 (50)	IM (*n* = 1)	IM (*n* = 1)
				NM (*n* = 5)	NM (*n* = 5)
		Non-concordant (%)	6 (50)	IM (*n* = 2)	NM (*n* = 2)
				NM (*n* = 1)	PM (*n* = 1)
				NM (*n* = 2)	UM (*n* = 2)
				IM (*n* = 1)	UM (*n* = 1)
CYP3A5	3	Concordant (%)	1 (33.3)	PM (*n* = 1)	PM (*n* = 1)
		Non-concordant (%)	2 (66.7)	PM (*n* = 1)	UM (*n* = 1)
				IM (*n* = 1)	PM (*n* = 1)

^a^ Number of cases that underwent simultaneous genotyping and phenotyping for the concerned isoenzyme; poor metabolizer (PM), intermediate metabolizer (IM), normal metabolizer (NM), rapid metabolizer (RM), and ultra-rapid metabolizer (UM).

**TABLE 3:** Comedication in patients undergoing concomitant genotyping and phenotyping.

ID internal number^a^	Drug of interest^b^	Inhibitory or inducing comedications^c^	Phenoconversion	Involved CYP	Drug-induced phenoconversion
20091212	Phenytoin, valproate	Clobazam, phenytoin, valproate	Yes	2C9: NM to UM	Yes: phenytoin (2C9)
20091552	No drug (familial history)	-	Yes	2D6: IM to NM	No
20091553	Dextromethorphan	-	No		
20092046	Various psychotropic agents	-	No		
20120141	Carbamazepine	Carbamazepine	Yes	2C19: IM to NM	No
20120963	Voriconazole	Voriconazole, cotrimoxazole	Yes	2C19: RM to NM	Yes: voriconazole (2C19)
				2D6: NM to PM	
20130518	Tacrolimus, everolimus	-	Yes	3A5: IM to PM	No
20151640	Risperidone	Risperidone, valproate	Yes	2D6: NM to UM	No
20160106	Various drugs	-	Yes	2C19: NM to PM	No
20160654	Phenytoin	Valproate, omeprazole, phenytoin	Yes	2C19: NM to UM	Yes: phenytoin (2C19)
20160995	Quetiapine	-	Yes	2D6: IM to UM	No
20160996	Risperidone	Risperidone	No		
20160997	Risperidone	Risperidone, escitalopram	No		
20161184	Risperidone	Risperidone	Yes	2D6: IM to NM	No
20171263	Drug-drug interaction between vemurafenib and immunosuppressants	Cotrimoxazole	Yes	2C19: UM to IM	No
				2D6: NM to UM	
20171269	Clonidine	Esomeprazole, risperidone, metronidazole	No		
20171688	Acenocoumarol	-	No		
20172044	Tacrolimus, ciclosporin	Ciclosporin, esomeprazole, cotrimoxazole, rifampicin	Yes	3A5: PM to UM	Yes: rifampicin (3A5)

^a^ Corresponds to our internal database number for the involved patient and consultation.

^b^ The drug because of which the genotyping/phenotyping test was performed.

^c^ According to our table of drug interaction, cytochrome P450 and P-glycoprotein (Samer et al., 2013).

Of all patients genotyped (*n* = 17 + 18 = 35), the most frequently investigated enzyme was CYP2D6 (*n* = 21), followed by CYP2C19 (*n* = 14) and CYP3A5 (*n* = 11).

Of all the patients who underwent phenotyping (*n* = 13 + 18 = 31), most received targeted phenotyping (*n* = 19) i.e., focusing on one, two, or eventually three CYP enzymes. Twelve patients received the full Geneva cocktail. The most frequently investigated enzyme was CYP3A4/5 (*n* = 27) followed by CYP2D6 (*n* = 23), particularly in the case of targeted phenotyping (CYP3A4/5, *n* = 16; CYP2D6, *n* = 11).

In most cases, investigations were carried out because of insufficient clinical response to treatment, low plasma concentrations or ADR. Half of the situations were related to immunosuppressive or antipsychotic treatments. In the other cases, analgesic, antiepileptic, antithrombotic, and antifungal drugs were involved. The 13 children (27%) on immunosuppressive therapy were mainly treated with tacrolimus (9/13) and were aged between 1 and 15 years. Eleven investigations were related to an antipsychotic treatment (risperidone or quetiapine). With the exception of one child aged 8 years, all others were adolescents aged 13 years or older.

Interestingly, genotyping or phenotyping results explained or contributed to the clinical event in 56% of cases (*n* = 27). In more than half of these cases (*n* = 15), the occurrence of ADR or high drug levels were the reasons for performing the tests. The involved drugs were mainly immunosuppressants followed by opioids and anticonvulsants. In the remaining 12 cases, insufficient therapeutic response or low drugs concentrations were the reasons. The involved drugs were mainly antipsychotics followed by immunosuppressants.

## Discussion

We reported our two decades of experience with CYP phenotyping or genotyping in children in a clinical setting. We performed these investigations because of insufficient clinical response to treatment, low plasma concentrations or ADR. Phenotyping and/or genotyping explained or contributed to these clinical events in more than half of cases.

The paediatric population has two specific features not present in adults: growth and maturation. Both are characterized by non-linear patterns from birth to adulthood and wide inter-individual variability (Kearns et al., 2003). Maturation and developmental changes have an impact on all stages of drug pharmacokinetics and pharmacodynamics (Kearns et al., 2003; van den Anker, 2010; Allegaert et al., 2017). Therefore, the paediatric population is highly heterogeneous, ranging from newborns under 1 kg with immature organs to adolescents over 100 kg. Effective and safe drug therapy can be challenging in such a heterogeneous population. Individualized dosage or medication according to patient characteristics is particularly relevant in this population (Lu and Rosenbaum, 2014). Individualization must take into account gender, weight, comorbidities but also pharmacogenomics and the impact of age on drug pharmacokinetics (McLaughlin et al., 2019).

CYPs metabolize 70%–80% of drugs and variations in their activity is a well-known factor of inter- and intra-individual variability in drug response. CYP activity can be modified by many factors; not only by concomitant xenobiotics administration but also by genetic polymorphism and age-related dependent changes in gene expression. CYP maturation is probably the predominant factor in age-associated pharmacokinetic differences between children and adults (de Wildt et al., 2014). Most CYPs have low activity at birth and reach adult levels between a few months and the first years of life with a different maturation profile (Kearns et al., 2003; Stevens et al., 2008; Allegaert and van den Anker, 2019). A poor ability to metabolize drugs can lead to accumulation and toxicity, and on the contrary, increased metabolization can lead to increased clearance and inefficacy. On the opposite, for pro-drugs, a reduced activity of a DME will lead to a loss of efficacy; and increased activity will result in a risk of toxicity.

Determining DME activity to guide therapy is one of the goals of personalized medicine. Gene-drug pairs with genetics-based prescribing guidelines are already being used in adults to guide current treatment (reactive testing) or to guide future treatment decisions (pre-emptive testing) (Whirl-Carrillo et al., 2012; Abdullah-Koolmees et al., 2020). Organizations such as the Clinical Pharmacogenetics Implementation Consortium (CPIC; https://cpicpgx.org/) (Relling and Klein, 2011; Relling et al., 2020), the Dutch Pharmacogenetics Working Group (DPWG) established by the Royal Dutch Pharmacist’s Association (KNMP; https://www.knmp.nl/) (Swen et al., 2008; Swen et al., 2011), the Pharmacogenomics Research Network (Shuldiner et al., 2013; Giacomini et al., 2021), and Ubiquitous Pharmacogenomics (Manson et al., 2017) have released easily accessible public guidelines for implementing pharmacogenetics in clinical practice.

Evidence-based pharmacogenomic testing in children is less robust (Sing et al., 2015; Hoshitsuki et al., 2021) and still little used in paediatrics. Most of the current guidelines, both for interpretation and implementation, are mainly based on adult data. A limited number of molecules, such as voriconazole and atomoxetine, have specific recommendations for children. Gene-drug associations applicable to children continue to be discovered (Ramsey et al., 2020; Brown et al., 2021). In a retrospective review, Roberts et al. estimated that pharmacogenetics could assist in treatment selection or dosing in almost half of the paediatric patients at an academic Children’s hospital (Roberts et al., 2021). Ramsay et al. (2020) found that in children, the annual prevalence of exposure to a drug with CPIC pharmacogenetics dosing recommendation was over 8′000 per 100′000 paediatric patients. Both studies found that CYP2D6, CYP2C19 and, to a lesser extent, CYP3A5 were the CYPs most frequently affected by actionable guidelines. Some institutions, such as the St. Jude Children’s Research Hospital in Memphis, have already clinically implemented pharmacogenetic testing and perform preemptive genotyping to guide and ensure drug prescribing for children (Hoffman et al., 2014; Gammal et al., 2016). CYP2D6 genotyping is thus successfully used to ensure safe prescription of codeine in children with pain in the context of sickle cell disease (Hoffman et al., 2014; Gammal et al., 2016).

Apart from being invasive, genotyping is easily performed in children of all ages. Genotyping results are immutable and independent of age. However, it does not consider the effect of gene ontogeny. The effect of a genotype variant depends on the expression of the gene. For this reason, in young children, depending on the developmental profile of the DME, the effect of the genotype may not be apparent (Ward et al., 2010; Leeder and Kearns, 2012; Hoshitsuki et al., 2021). Another limitation of genotyping is that it must interrogate all known alleles in the patient population of interest. Finally, at the time of our study, genotyping was also limited by its high cost and reimbursement concerns by the health insurance. Although since 2017 this is no longer a problem in Switzerland, the issue of price and reimbursement must be kept in mind (Shah and Smith, 2015).

Phenotyping combines the effects of genetic, environmental, and developmental factors on CYP activity, and it therefore makes it possible to consider phenoconversion. It allows better prediction of the CYP activity at a given time point. However, it requires the administration of specific and non-toxic probes to children, followed by analysis of parent drugs and metabolites in biofluids. Validated MR interpretations are needed, in particular in children. The acceptability of oral probes in young children can be challenging (Magliocco and Daali, 2020; Magliocco et al., 2020). Thus, administration of a single capsule with multiple substrates and fixed doses, such as the Geneva cocktail used in adults, is difficult in children because of the risk of toxicity. In young children, a selective phenotyping approach by administration of a targeted probe with an individualized dosage adapted to age and weight is essential. Finally, there is a need to develop sensitive analytical methods due to the reduced dosage and low plasma concentrations (Magliocco and Daali, 2020; Magliocco et al., 2020).

Our study showed a highly variable concordance between genotype and phenotype in children. The lack of concordance occurred regardless of the age of the children and was, in half of the cases, for CYP2C19 and CYP3A5, explained by co-medication. For CYP2D6, the low sensitivity of genotyping tests to detect UM may have contributed to the non-concordant cases (Rebsamen et al., 2009), as well as default genotype calls. Furthermore, challenges of wide, overlaping and non-validated ranges in children for phenotype classification have to been taken in consideration, especially in our study where the phenotyping methods have changed over the years. With the exception of PM, our study in adults also showed a poor correlation between the predicted and measured phenotype (Ing Lorenzini et al., 2021). The small size of our cohort prevents a definitive conclusion.

No investigations were done preemptively before the initiation of treatment. They were all carried out to understand unexpected responses to a drug, its ineffectiveness or toxicity. Our study showed that the results of metabolic investigations could contribute to the clinical event in up to 60% of clinical situations.

In conclusion, there is clearly value in a guided drug dosing based on CYP activity in children. However genetically based dosing algorithms used in adults cannot simply be extrapolated to young children, without considering the impact of ontogeny. Data describing the genotype-phenotype interaction in children are still scarce, particularly in sick and young children. Our study shows that genotyping and phenotyping are two complementary methods that can be used in children to determine CYP activity. The choice to perform genotyping, phenotyping or both should be considered on a case-by-case basis, taking into account their respective limitations, the clinical question and the patient. When used appropriately, both methods are helpful to understand interpatient variability in drug exposure and response, but probably also for guiding future treatments (pre-emptive testing).

## Data Availability

The data analyzed in this study is subject to the following licenses/restrictions: The data that support the findings of this study are available on request from the corresponding author, FR. The data are not publicly available as they contain information that could compromise the privacy of research participants. Requests to access these datasets should be directed to frederique.rodieux@hcuge.ch.

## References

[B1] Abdullah-Koolmees H. van Keulen A. M. Nijenhuis M. Deneer V. H. M. (2020). Pharmacogenetics guidelines: Overview and comparison of the DPWG, CPIC, CPNDS, and RNPGx guidelines. Front. Pharmacol. 11, 595219. 10.3389/fphar.2020.595219 33568995 PMC7868558

[B2] Allegaert K. Mian P. van den Anker J. N. (2017). Developmental pharmacokinetics in neonates: Maturational changes and beyond. Curr. Pharm. Des. 23 (38), 5769–5778. 10.2174/1381612823666170926121124 28950819

[B3] Allegaert K. van den Anker J. (2019). Ontogeny of phase I metabolism of drugs. J. Clin. Pharmacol. 59 (1), S33–s41. 10.1002/jcph.1483 31502685

[B4] Baumann P. Amstutz U. Bühlmann R. P. Meier-Abt P. Meyer U. A. Samer C. (2017). Pharmacogénomique et thérapie personnalisée. Rev. Med. Suisse 13 (573), 1544–1545. 10.53738/revmed.2017.13.573.1544 28876714

[B5] Bosilkovska M. Samer C. F. Déglon J. Rebsamen M. Staub C. Dayer P. (2014). Geneva cocktail for cytochrome p450 and P-glycoprotein activity assessment using dried blood spots. Clin. Pharmacol. Ther. 96 (3), 349–359. 10.1038/clpt.2014.83 24722393 PMC4151019

[B6] Brown J. T. Ramsey L. B. Van Driest S. L. Aka I. Colace S. I. (2021). Characterizing pharmacogenetic testing among Children's hospitals. Clin. Transl. Sci. 14 (2), 692–701. 10.1111/cts.12931 33325650 PMC7993279

[B7] de Wildt S. N. Tibboel D. Leeder J. S. (2014). Drug metabolism for the paediatrician. Arch. Dis. Child. 99 (12), 1137–1142. 10.1136/archdischild-2013-305212 25187498

[B8] FOPH (2018). Swiss Federal Office of Public Health. Loi fédérale sur l'analyse génétique humaine (LAGH) [Online]. Available: https://www.fedlex.admin.ch/eli/cc/2022/537/fr (Accessed January, 2023).

[B9] Gaedigk A. Ingelman-Sundberg M. Miller N. A. Leeder J. S. Whirl-Carrillo M. Klein T. E. (2018). The Pharmacogene variation (PharmVar) consortium: Incorporation of the human cytochrome P450 (CYP) allele nomenclature database. Clin. Pharmacol. Ther. 103 (3), 399–401. 10.1002/cpt.910 29134625 PMC5836850

[B10] Gaedigk A. Sangkuhl K. Whirl-Carrillo M. Twist G. P. Klein T. E. Miller N. A. (2019). The evolution of PharmVar. Clin. Pharmacol. Ther. 105 (1), 29–32. 10.1002/cpt.1275 30536702 PMC6312487

[B11] Gammal R. S. Crews K. R. Haidar C. E. Hoffman J. M. Baker D. K. Barker P. J. (2016). Pharmacogenetics for safe codeine use in sickle cell disease. Pediatrics 138 (1), e20153479. 10.1542/peds.2015-3479 27335380 PMC4925073

[B12] Giacomini K. M. Karnes J. H. Crews K. R. Monte A. A. Murphy W. A. Oni-Orisan A. (2021). Advancing precision medicine through the new pharmacogenomics global research Network. Clin. Pharmacol. Ther. 110 (3), 559–562. 10.1002/cpt.2340 34318925 PMC8881968

[B13] Goh L. L. Lim C. W. Sim W. C. Toh L. X. Leong K. P. (2017). Analysis of genetic variation in CYP450 genes for clinical implementation. PLoS One 12 (1), e0169233. 10.1371/journal.pone.0169233 28046094 PMC5207784

[B14] Hoffman J. M. Haidar C. E. Wilkinson M. R. Crews K. R. Baker D. K. Kornegay N. M. (2014). PG4KDS: A model for the clinical implementation of pre-emptive pharmacogenetics. Am. J. Med. Genet. C Semin. Med. Genet. 166c (1), 45–55. 10.1002/ajmg.c.31391 24619595 PMC4056586

[B15] Hoshitsuki K. Fernandez C. A. Yang J. J. (2021). Pharmacogenomics for drug dosing in children: Current use, knowledge, and gaps. J. Clin. Pharmacol. 61 (1), S188–s192. 10.1002/jcph.1891 34185912 PMC8259917

[B16] Ing Lorenzini K. Desmeules J. Rollason V. Bertin S. Besson M. Daali Y. (2021). CYP450 genotype-phenotype concordance using the Geneva micrococktail in a clinical setting. Front. Pharmacol. 12, 730637. 10.3389/fphar.2021.730637 34512355 PMC8427306

[B17] Kearns G. L. Abdel-Rahman S. M. Alander S. W. Blowey D. L. Leeder J. S. Kauffman R. E. (2003). Developmental pharmacology--drug disposition, action, and therapy in infants and children. N. Engl. J. Med. 349 (12), 1157–1167. 10.1056/NEJMra035092 13679531

[B18] Leeder J. S. Kearns G. L. (2012). Interpreting pharmacogenetic data in the developing neonate: The challenge of hitting a moving target. Clin. Pharmacol. Ther. 92 (4), 434–436. 10.1038/clpt.2012.130 22948896

[B19] Lloret-Linares C. Rollason V. Lorenzini K. I. Samer C. Daali Y. Gex-Fabry M. (2017). Screening for genotypic and phenotypic variations in CYP450 activity in patients with therapeutic problems in a psychiatric setting, a retrospective study. Pharmacol. Res. 118, 104–110. 10.1016/j.phrs.2016.07.002 27378571

[B20] Lu H. Rosenbaum S. (2014). Developmental pharmacokinetics in pediatric populations. J. Pediatr. Pharmacol. Ther. 19 (4), 262–276. 10.5863/1551-6776-19.4.262 25762871 PMC4341411

[B21] Magliocco G. Daali Y. (2020). Modern approaches for the phenotyping of cytochrome P450 enzymes in children. Expert Rev. Clin. Pharmacol. 13 (7), 671–674. 10.1080/17512433.2020.1779057 32500746

[B22] Magliocco G. Rodieux F. Desmeules J. Samer C. F. Daali Y. (2020). Toward precision medicine in pediatric population using cytochrome P450 phenotyping approaches and physiologically based pharmacokinetic modeling. Pediatr. Res. 87 (3), 441–449. 10.1038/s41390-019-0609-z 31600772

[B23] Manikandan P. Nagini S. (2018). Cytochrome P450 structure, function and clinical significance: A review. Curr. Drug Targets 19 (1), 38–54. 10.2174/1389450118666170125144557 28124606

[B24] Manson L. E. van der Wouden C. H. Swen J. J. Guchelaar H. J. (2017). The ubiquitous pharmacogenomics consortium: Making effective treatment optimization accessible to every European citizen. Pharmacogenomics 18 (11), 1041–1045. 10.2217/pgs-2017-0093 28685652

[B25] McLaughlin M. J. Wagner J. Shakhnovich V. Carleton B. Leeder J. S. (2019). Considerations for implementing precision therapeutics for children. Clin. Transl. Sci. 12 (2), 140–150. 10.1111/cts.12607 30516322 PMC6440566

[B26] Nimmagadda S. Wong S. J. Faria M. Allen P. Faria J. (2020). Potential cytochrome P450 drug-drug interaction among adult and adolescent patients undergoing tonsillectomy. OTO Open 4 (2), 2473974x20932503. 10.1177/2473974x20932503 PMC7303781 32596625

[B27] Ogu C. C. Maxa J. L. (2000). Drug interactions due to cytochrome P450. Proc. (Bayl Univ. Med. Cent. 13 (4), 421–423. 10.1080/08998280.2000.11927719 16389357 PMC1312247

[B28] Ramsey L. B. Ong H. H. Schildcrout J. S. Shi Y. Tang L. A. Hicks J. K. (2020). Prescribing prevalence of medications with potential genotype-guided dosing in pediatric patients. JAMA Netw. Open 3 (12), e2029411. 10.1001/jamanetworkopen.2020.29411 33315113 PMC7737091

[B29] Rebsamen M. C. Desmeules J. Daali Y. Chiappe A. Diemand A. Rey C. (2009). The AmpliChip CYP450 test: Cytochrome P450 2D6 genotype assessment and phenotype prediction. Pharmacogenomics J. 9 (1), 34–41. 10.1038/tpj.2008.7 18591960

[B30] Relling M. V. Klein T. E. (2011). CPIC: Clinical pharmacogenetics implementation consortium of the pharmacogenomics research Network. Clin. Pharmacol. Ther. 89 (3), 464–467. 10.1038/clpt.2010.279 21270786 PMC3098762

[B31] Relling M. V. Klein T. E. Gammal R. S. Whirl-Carrillo M. Hoffman J. M. Caudle K. E. (2020). The clinical pharmacogenetics implementation consortium: 10 Years later. Clin. Pharmacol. Ther. 107 (1), 171–175. 10.1002/cpt.1651 31562822 PMC6925644

[B32] Roberts T. A. Wagner J. A. Sandritter T. Black B. T. Gaedigk A. Stancil S. L. (2021). Retrospective review of pharmacogenetic testing at an academic Children's hospital. Clin. Transl. Sci. 14 (1), 412–421. 10.1111/cts.12895 33048453 PMC7877836

[B33] Rollason V. Lloret-Linares C. Lorenzini K. I. Daali Y. Gex-Fabry M. Piguet V. (2020a). Evaluation of phenotypic and genotypic variations of drug metabolising enzymes and transporters in chronic pain patients facing adverse drug reactions or non-response to analgesics: A retrospective study. J. Pers. Med. 10 (4), 198. 10.3390/jpm10040198 33121061 PMC7711785

[B34] Rollason V. Mouterde M. Daali Y. Čížková M. Priehodová E. Kulichová I. (2020b). Safety of the Geneva cocktail, a cytochrome P450 and P-glycoprotein phenotyping cocktail, in healthy volunteers from three different geographic origins. Drug Saf. 43 (11), 1181–1189. 10.1007/s40264-020-00983-8 32851583 PMC7575470

[B35] Samer C. F. Lorenzini K. I. Rollason V. Daali Y. Desmeules J. A. (2013). Applications of CYP450 testing in the clinical setting. Mol. Diagn Ther. 17 (3), 165–184. 10.1007/s40291-013-0028-5 23588782 PMC3663206

[B36] Shah R. R. Smith R. L. (2015). Addressing phenoconversion: The achilles' heel of personalized medicine. Br. J. Clin. Pharmacol. 79 (2), 222–240. 10.1111/bcp.12441 24913012 PMC4309629

[B37] Shuldiner A. R. Relling M. V. Peterson J. F. Hicks J. K. Freimuth R. R. Sadee W. (2013). The pharmacogenomics research Network translational pharmacogenetics program: Overcoming challenges of real-world implementation. Clin. Pharmacol. Ther. 94 (2), 207–210. 10.1038/clpt.2013.59 23588301 PMC3720847

[B38] Sing C. W. Cheung C. L. Wong I. C. (2015). Pharmacogenomics--how close/far are we to practising individualized medicine for children? Br. J. Clin. Pharmacol. 79 (3), 419–428. 10.1111/bcp.12338 25855823 PMC4345952

[B39] Stevens J. C. Marsh S. A. Zaya M. J. Regina K. J. Divakaran K. Le M. (2008). Developmental changes in human liver CYP2D6 expression. Drug Metab. Dispos. 36 (8), 1587–1593. 10.1124/dmd.108.021873 18474679

[B40] Swen J. J. Nijenhuis M. de Boer A. Grandia L. Maitland-van der Zee A. H. Mulder H. (2011). Pharmacogenetics: From bench to byte--an update of guidelines. Clin. Pharmacol. Ther. 89 (5), 662–673. 10.1038/clpt.2011.34 21412232

[B41] Swen J. J. Wilting I. de Goede A. L. Grandia L. Mulder H. Touw D. J. (2008). Pharmacogenetics: From bench to byte. Clin. Pharmacol. Ther. 83 (5), 781–787. 10.1038/sj.clpt.6100507 18253145

[B42] van den Anker J. N. (2010). Developmental pharmacology. Dev. Disabil. Res. Rev. 16 (3), 233–238. 10.1002/ddrr.122 20981761

[B43] Volpi S. Bult C. J. Chisholm R. L. Deverka P. A. Ginsburg G. S. Jacob H. J. (2018). Research directions in the clinical implementation of pharmacogenomics: An overview of US programs and projects. Clin. Pharmacol. Ther. 103 (5), 778–786. 10.1002/cpt.1048 29460415 PMC5902434

[B44] Ward R. M. Tammara B. Sullivan S. E. Stewart D. L. Rath N. Meng X. (2010). Single-dose, multiple-dose, and population pharmacokinetics of pantoprazole in neonates and preterm infants with a clinical diagnosis of gastroesophageal reflux disease (GERD). Eur. J. Clin. Pharmacol. 66 (6), 555–561. 10.1007/s00228-010-0811-8 20306184

[B45] Whirl-Carrillo M. McDonagh E. M. Hebert J. M. Gong L. Sangkuhl K. Thorn C. F. (2012). Pharmacogenomics knowledge for personalized medicine. Clin. Pharmacol. Ther. 92 (4), 414–417. 10.1038/clpt.2012.96 22992668 PMC3660037

[B46] Zhou S. F. Liu J. P. Chowbay B. (2009). Polymorphism of human cytochrome P450 enzymes and its clinical impact. Drug Metab. Rev. 41 (2), 89–295. 10.1080/03602530902843483 19514967

